# Carbon Nanohorn Suprastructures on a Paper Support as a Sorptive Phase

**DOI:** 10.3390/molecules23061252

**Published:** 2018-05-24

**Authors:** Julia Ríos-Gómez, Beatriz Fresco-Cala, María Teresa García-Valverde, Rafael Lucena, Soledad Cárdenas

**Affiliations:** Departamento de Química Analítica, Instituto Universitario de Investigación en Química Fina y Nanoquímica IUIQFN, Universidad de Córdoba, Campus de Rabanales, Edificio Marie Curie (anexo), E-14071 Córdoba, Spain; juliariosgomez@hotmail.com (J.R.-G.); q72frcab@uco.es (B.F.-C.); q72gavam@uco.es (M.T.G.-V.); scardenas@uco.es (S.C.)

**Keywords:** carbon nanohorns, sorptive phase, paper, microextraction, antidepressants

## Abstract

This article describes a method for the modification of paper with single-wall carbon nanohorns (SWCNHs) to form stable suprastructures. The SWCNHs form stable dahlia-like aggregates in solution that are then self-assembled into superior structures if the solvent is evaporated. Dipping paper sections into a dispersion of SWCNHs leads to the formation of a thin film that can be used for microextraction purposes. The coated paper can be easily handled with a simple pipette tip, paving the way for disposable extraction units. As a proof of concept, the extraction of antidepressants from urine and their determination by direct infusion mass spectrometry is studied. Limits of detection (LODs) were 10 ng/L for desipramine, amitriptyline, and mianserin, while the precision, expressed as a relative standard deviation, was 7.2%, 7.3%, and 9.8%, respectively.

## 1. Introduction

Solid-phase microextraction (SPME) is a consolidated sample treatment technique that combines isolation, preconcentration, and sample introduction into one step [1]. This miniaturized technique, which can easily be automated, is based on the distribution of the analytes between the sample and the fiber coating. In this context, the reversible chemical interactions between the analyte and the sorptive phase are of paramount importance to define the efficiency and selectivity of the microextraction. SPME is in a continuous development following several evident tendencies like the development of new coatings [2,3] or the direct coupling with instrumental techniques like mass-spectrometry (MS) [4,5]. All these trends make SPME the predominant technology for microextraction.

The adaptation of the SPME principles to a specific field such as environmental analysis drove the development of thin film microextraction (TFME) [6]. Although both techniques share the same foundations, they differ in their application. TFME uses a thin sheet of a polymeric phase as a sorptive phase that may adopt several shapes [7] like a flat membrane [8] or a coated blade [9]. These formats present a higher extraction capacity compared to traditional fibers due to an increased surface to volume ratio, which has positive connotations for the thermodynamic and kinetics. Also, TFME can be automated to allow the simultaneous extraction of several samples, thus increasing the sample throughput [10]. In the typical procedure, the thin film is immersed in the sample, which is stirred to favor the analyte transference to the sorptive phase [11]. However, the thin film can also be stirred into the sample. The use of planar sorptive phases integrated into stirring units has allowed for the development of new techniques like rotating disk sorptive extraction [12] and stir membrane extraction [13,14].

As with SPME, the development of new sorptive phases is crucial in TFME. Fortunately, there is a wide range of materials that can be used, from commercial membranes to lab-made materials. Among the latter, fabric phases and electrospun membranes can be highlighted. Fabric phases, first proposed by Kabir and Furton, consist of the chemical modification by sol-gel reaction of fabrics (cotton, glass fiber) to introduce functional polymers to its surface [15]. On the other hand, electrospun membranes provide the analyst with a wide range of tools since the characteristics of the final product depend on the polymeric precursor(s) and conditions used during the electrospinning [16]. The use of nanoparticles (NPs) as ingredients in these materials makes their application scope even broader. The presence of these NPs usually enhances the sorption capacity by two mechanisms that can be complementary: NPs may introduce new sorption sites in the polymer structure, and may increase the superficial area of the polymer [17].

A NP can be defined as a particle that has at least one dimension in the nanometric range (100 nm is used as a limit by convention) and presents unique properties (not observable in the bulk material) because of its size [18]. There would be a myriad of different NPs if we considered their chemical composition, size, shapes, and potential combinations. From a chemical point of view, NPs can be classified as inorganic or carbon-based. The latter, including fullerenes and nanotubes, have been extensively used in microextraction [19], although the use of single-walled carbon nanohorns (SWCNHs), first described by Ijima et al. in 1991 [20], is limited [21,22]. SWCNHs consist of horn-shaped sheath aggregates of graphene. They usually present lengths in the range of 40–50 nm and an inner diameter from 2 to 5 nm. Their oxidation, to introduce oxygen-containing functional groups to the surface, is easier compared to carbon nanotubes. In solution, SWCNHs are prone to aggregate, forming ordered and stable structures called dahlias [23]. Although the aggregation tendency is common for all carbon nanoparticles, especially when their surface is not chemically modified, these ordered aggregates have been only described for CNHs. This particularity is exploited in the present work.

Oakes et al. proposed in 2013 the electrodeposition of CNHs in different substrates, opening the door for the synthesis of CNHs-coated materials for catalysis, sensing, or energy storage applications [24]. In this article, we propose for the first time the use of SWCNHs-modified paper as a sorptive phase in TFME without the assistance of an anchoring polymer. It has been observed that the dahlia aggregates, obtained in solution, form suprastructures (ordered combinations of single dahlias) when the solvent is evaporated. These suprastructures present a porous conformation that enhances their sorption ability. To make the handling of this sorptive phase easier, it has been coated over conventional paper. It is necessary to indicate that paper acts as a simple support and that suprastructures form over its surface. A small percentage of the suprastructure is embedded in the cellulose fibers, thus improving the mechanical stability of the sorptive phase.

The use of paper as a support opens the door for the development of cheap and disposable units. Meng et al. proposed the use of unmodified paper for the extraction of 8-hydroxy-2′-deoxyguanosine from a urine sample [25], while Saraji and Farajmand have reported the use of modified paper as a sorptive phase [26]. Our research group proposed the direct coating of conventional paper with polymers as a simple strategy to synthesize new sorptive phases [27]. Also, the resulting sorptive phase can be easily cut to the desired shape and length. The potential of paper goes beyond these applications since it can be directly analyzed by MS, in the so-called paper spray MS [28,29,30,31], which implies a dramatic simplification of the analytical process. To be used in the paper spray mode, the phases must be conductive and mechanically stable to avoid MS source contamination. As the mechanical stability of this phase in a high-voltage gradient should be previously guaranteed, this initial research will be focused on the evaluation of its sorption ability towards selected antidepressants in urine. To speed up and simplify the analysis, the eluates are directly infused [32,33] in the mass spectrometer, thereby avoiding a previous chromatographic separation.

## 2. Materials and Methods

### 2.1. Reagents

All reagents were of analytical grade or better. Unless otherwise specified, they were purchased from Sigma Chemical Co. (St. Louis, MO, USA, https://www.sigmaaldrich.com/). Stock standard solutions of the antidepressants (mianserin, trimipramine, desipramine, and amitriptyline) were prepared in methanol at a concentration of 1 g/L and stored at 4 °C. Working standard solutions were prepared daily by rigorous dilution of the stocks in ultrapure Milli-Q water. Methanol:acetic acid (95:5) was also used for antidepressants elution after the extraction. Deuterated 5-hydroxyindole-3-acetic acid (5-HIAA-D5) was used as an internal standard for MS measurements. The working concentration of the internal standard was 100 ng/mL.

SWCNHs were purchased from Carbonium S.r.l. (Padua, Italy, www.carbonium.it/). They form stable dahlia-shaped aggregates with an average diameter of 60–80 nm. Individually, the lengths of these SWCNHs are in the range 40–50 nm, and the width in the cylindrical structure varies between 4 and 5 nm. For the synthesis of the sorptive phases, SWCNTs were dispersed in chloroform.

Acetonitrile, acetic acid (Scharlab, Barcelona, Spain, http://www.scharlab.com/), triethylamine, and ultrapure Milli-Q water were employed as components of the chromatographic mobile phase.

Blank urine samples were collected from healthy adult volunteers and stored in polytetrafluoroethylene (PTFE) flasks at −20 °C until analysis. Before the extraction, each sample was 1:1 diluted with ultrapure water and the pH was also adjusted to 10 with sodium hydroxide. The pH is fixed at alkaline conditions to promote the interaction between the basic analytes with the sorptive phase. The interaction between the sorptive phase and the analytes (their chemical structures and the logarithm of the octanol/water partition coefficients are shown in Figure S1) involve general hydrophobic interactions and π–π bonds with the aromatic moieties. The samples are not filtered before their extraction.

### 2.2. Synthesis and Characterization of Sorptive Phases

The synthesis follows a simple workflow. First, 10 mg of SWCNHs are dispersed by manual shaking in 150 µL of chloroform inside an Eppendorf flask. Once dispersed, segments of filter paper (3 × 0.5 cm) are dipped three consecutive times into the dispersion, drying the paper after each dip. The evaporation of the solvent leaves a suprastructure of dahlia aggregates over the paper surface. The resulting materials were characterized by scanning electron microscopy (SEM) using a JEOL JSM 7800 microscope (JEOL, Tokyo, Japan). Micrographs were acquired at the central Service for Research Support (SCAI) of the University of Córdoba.

### 2.3. Microextraction Procedure

**Figure 1:**
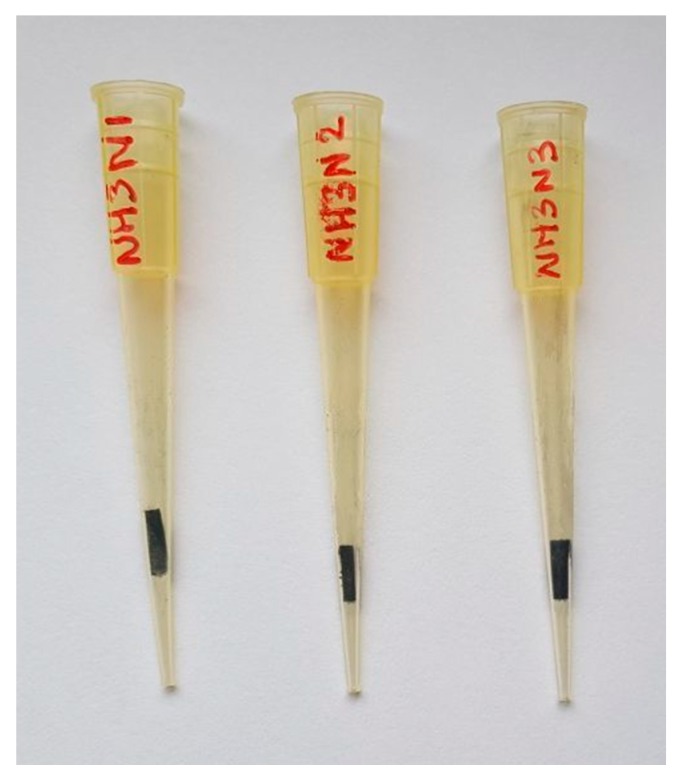
Three different pipette tip extraction units containing carbon nanohorn suprastructures coated over conventional paper as a sorptive phase. The phase is mechanically fixed to the narrower section of the tip and in close contact with its inner walls.

A plastic pipette is used to build a simple extraction device. A segment of sorptive phase with an area of 0.25 cm^2^ is located and mechanically fixed (physically caught in the narrower section) in a 200 µL pipette tip, as indicated in Figure 1. The extraction process comprises several steps. Initially, 1200 µL of the pretreated sample are located in a vial. In the meantime, the sorptive phase is conditioned with 200 µL of methanol and 200 µL of an alkaline aqueous solution (pH = 10), which are aspirated and ejected. Once the sorptive phase is in the best conditions, the tip is immersed in the sample vial, and 80 strokes (aspiration and ejection cycles) are performed to maximize the interaction of the analytes with the membrane. Before the final elution of the analytes using 50 µL of methanol:acetic acid (95:5, *v/v*), the sorptive phase is washed with 200 µL of alkaline aqueous solution (pH = 10). The extracts are analyzed by UPLC-DAD or direct infusion MS for analyte identification and quantification, as indicated in the next section. For direct infusion MS, the internal standard is added to the eluent at a concentration of 100 ng/mL.

### 2.4. Instrumental Techniques

Two instrumental techniques were employed in the development of the present research. The optimization of the extraction procedure and its preliminary analytical evaluation was carried out on a Waters AcquityTM Ultra Performance LC system (Waters Corp., Madrid, Spain) using an Acquity UPLC^®^ BEH C18 column (1.7 μm, 2.1 mm × 100 mm) working at the experimental conditions described in the Supplementary Materials. Direct infusion MS measurements were performed on Agilent 6420 Triple Quadrupole MS with electrospray source using Agilent MassHunter Software (version B.06.00, Santa Clara, CA, USA) for data analyses. The mass spectrometer settings were fixed to improve the SRM signal. The flow rate and the temperature of the drying gas (N_2_) were 9 L/min and 300 °C, respectively. The nebulizer pressure was 40 psi, and the capillary voltage was kept to 2000 V in positive mode. The analytes and the internal standard were detected by Selected Reaction Monitoring (SRM) transitions, the parameters being specified in Table S1.

## 3. Results and Discussion

### 3.1. Synthesis and Characterization of Sorptive Phases

**Figure 2:**
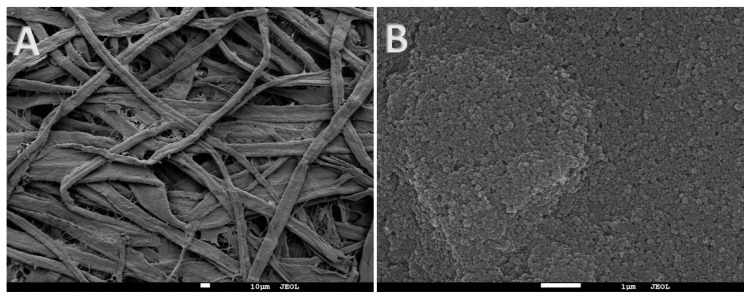
SEM micrographs of (**A**) unmodified paper (at 300 magnifications) and (**B**) coated paper (at 13,000× magnification). The presence of the SWNHs suprastructure, which completely covers the cellulose fibers, is observable on the surface.

Paper is an excellent support for the preparation of new microextraction and sensing platforms due to its low price, high porosity, and easy chemical modification. Conventional paper consists of natural cellulose fibers, mechanically compacted, creating a crisscross pattern, as can be observed in Figure 2A. On the other hand, SWCNHs aggregate in solution, forming a stable structure called a dahlia. When the solvent evaporates, these aggregates form suprastructures that consist of self-assembled dahlias. If an SWCNHs dispersion in chloroform is prepared, and a conventional paper is dipped into it, these suprastructures can be created after the solvent evaporation over the paper surface. Dipping, among other approaches, has been proposed for the preparation of coatings in SPME [34]. Figure 2B shows the SEM picture of modified paper where single nanometric dahlias, showing a semispherical shape, are easily identified, while the typical fibrous structure of the unmodified paper is not observed.

**Figure 3:**
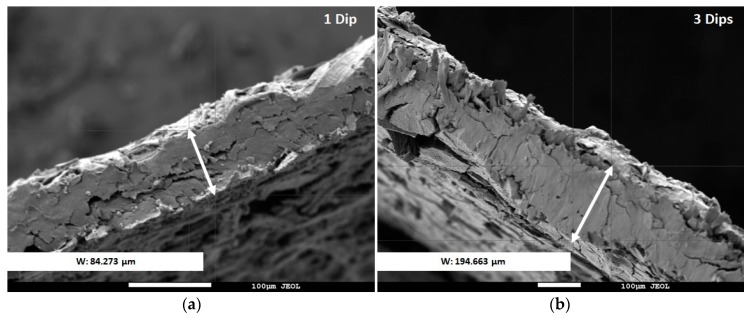
SEM micrographs of the side profile of sorptive phases synthesized after (**a**) one dip (at 270 magnifications) and (**b**) three dips (at 140 magnification). The thickness of the coating (considering the different scale of the pictures) increases from ca. 84 to 190 µm.

The thickness of the suprastructure coating can easily be modified by increasing the number of paper dips. Figure 3 shows the superficial SEM pictures of two sorptive phases fabricated using one and three dips, respectively. The thickness of the coating increases from ca. 84 to 190 µm. The thickness is directly related to the extraction capacity, as can be observed for desipramine in Figure S2. The best extraction recoveries are obtained for three dips, which indicates that the sorption is not only superficial; the size of the pores is sufficient for the diffusion of the analytes. Although the extraction is not exhaustive, the results are comparable with those obtained in other microextraction techniques.

It is assumed that the mechanical stability is the critical issue of the new phase. However, different tests have demonstrated acceptable stability under working conditions where the sample is passed laterally (it does not flow) through the sorptive phase. On the one hand, after the synthesis, the sorptive phases are cleaned with different solvents, and negligible detachment of the suprastructures is observed. Although the extracts are filtered before their injection in the UPLC, the dahlia sizes (60–80 nm) are smaller than the filter pore size (0.22 µm). A detachment of the nanoparticles would affect the column backpressure (which is very sensitive to the introduction of particles) in UPLC analyses. However, the pressure remained in the normal working range. Finally, sorptive phases can be reused up to 100 times, which indicates that the sorptive phase is not lost during the extraction.

The explanation of this stability can be found on the support. Paper is porous, and the first layer of CNHs aggregates can be occluded on its structure while the subsequent layers can be stabilized by non-covalent (π–π) interactions. This stabilization and the working conditions previously described can be the reason behind this acceptable stability.

### 3.2. Extraction Evaluation

The effect of three critical parameters on the extraction of the analytes was evaluated. The sensitivity and precision enhancement were considered to select the most appropriate conditions. The optimization was done following a one variable at a time approach. Once optimized, the variable is fixed at its optimum value to study the rest of the parameters. Each condition was evaluated in triplicate.

**Figure 4:**
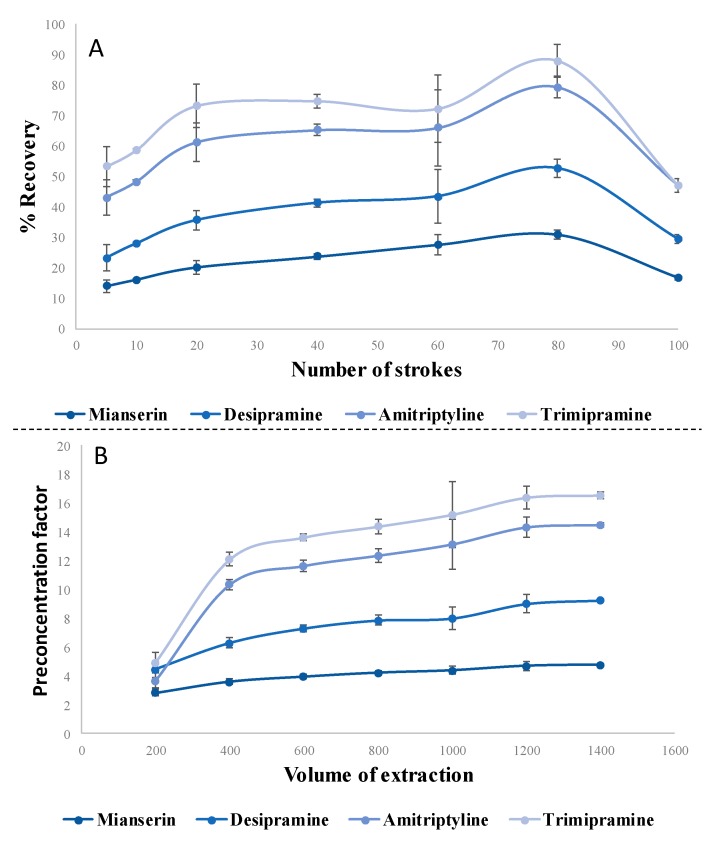
Effect of the (**A**) number of strokes and (**B**) sample volume on the extraction recovery of the analyte. The sample volume is defined as the volume of sample placed in the extraction vial. Each condition was evaluated in triplicate.

The number of sample strokes (times that the solution is pulled into and ejected from the pipette tip) was evaluated, placing 800 µL of an aqueous standard in a glass vial. As can be observed in Figure 4A, the extraction recovery increases with the number of strokes up to 80 cycles, before decreasing for further values. The number of cycles indicates that the diffusion of the analytes from the solution to the sorptive phases is not promoted. This fact can be ascribed to the geometry of the pipette tip and the location of the phase in one of the inner walls of the tip (see Figure 1), which permits the direct contact of only a fraction of the sample with the sorptive phase. The modification of the extraction unit to enhance this transference is a current line of research.

The sample volume was also considered in depth. This volume was defined as the volume of sample placed in the extraction vial. For the extraction, the pipette tip containing the sorptive phase is immersed in the sample, and 80 strokes are done without taking the tip out of the sample. As can be observed, the peak of the area of the analytes increased with the initial sample volume up to 1200 µL (Figure 4B), which was selected as the optimum value. This volume is compatible with bioanalytical samples like urine or saliva.

Finally, the number of elution strokes was considered. The results (data not shown) indicate that 10 cycles were enough to elute the analytes.

### 3.3. Analytical Evaluation

The combination of the extraction workflow with chromatographic analysis (UPLC-DAD) was initially considered to fully understand the potential of the sample treatment. For this purpose, a calibration curve for each analyte was constructed. Good linearity (*R* > 0.995) was observed in the range 10–1500 µg/L for desipramine, amitriptyline, and mianserin, while trimipramine presented a slightly lower value (*R* = 0.989). The limits of detection, which were calculated using a signal to noise ratio of 3, were in the range of 0.1 µg/L for desipramine, amitriptyline, and mianserin, while trimipramine presented a slightly higher value (0.2 µg/L). The repeatability of the method, expressed as the relative standard deviation (RSD, %), ranged from 3.8% (amitriptyline) to 7.4% (trimipramine). The analysis of a raw urine sample with the method did not show good performance, with relative recoveries (calculated at 200 µg/L) in the 50–60% range, although the 1:1 dilution of the sample fulfilled the 70–130% recovery criterion.

Once the performance of the extraction workflow in combination with UPLC-DAD was evaluated, its direct coupling with MS was also studied. Direct infusion MS allows the reduction of the analysis time, providing good selectivity (working in the SRM mode) and sensitivity (if ion-suppression is negligible). In most cases, ESI-MS requires the use of an internal standard to improve the precision measurement, and in this case, 5-HIAA-D5 was used for this purpose. According to the results obtained with the UPLC-UV combination, in-matrix calibration was selected for the direct infusion approach. Interestingly, the calibration curves obtained for the analytes in the range from 0.1 to 10 µg/L (six different concentration levels, *n* = 3), and prepared in blank urine 1:1 diluted in water, were linear (*R* > 0.993) for almost all the analytes, trimipramine excepted (*R* = 0.9), while the limits of detection were in the range of 10 ng/L for desipramine, amitriptyline, and mianserin. The precision, expressed as RSD values, was evaluated at 0.1 µg/L considering three replicates. The values of the last three analytes were 7.2%, 7.3%, and 9.8%, respectively.

Table 1 summarizes and compares the sensitivity levels provided by different analytical methods [35,36,37,38,39,40] proposed for the determination of antidepressant drugs in biological fluids. The new approach provided the best results thanks to the use of direct infusion MS as instrumental technique. This combination combines the inherent sensitivity of MS with the higher injection volumes allowed in direct infusion. In fact, the sample volume is limited in chromatographic separation by the resolution factor.

## 4. Conclusions

**Figure 5:**
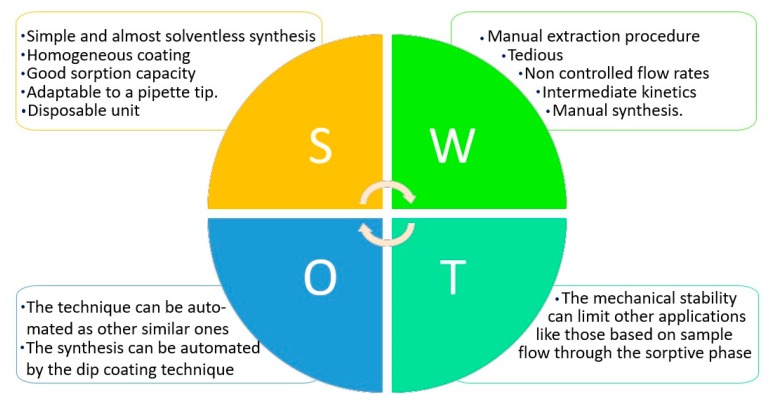
SWOT (Strengths, Weaknesses, Opportunities, and Threats) analysis of the new sorptive phase.

This article presents carbon nanohorn suprastructures coated over conventional paper as a sorptive phase in thin film microextraction. To make a critical and complete study of the new phase, a SWOT (Strengths, Weaknesses, Opportunities, and Threats) analysis has been done. This study, which is schematically presented in Figure 5, is focused on the new sorptive phase rather than the analytical application.

The synthesis is simple as it only requires dipping the paper into an organic dispersion containing the nanostructures. The evaporation of the solvent leaves a continuous and homogeneous layer of aggregated dahlias over the paper surface, which can interact with the target analytes. The volume of solvent is very low, and the synthesis can be considered almost solventless. The as-prepared sorptive phases, which have dimensions of 3 × 0.5 cm, are finally adapted to conventional pipette tips, which act as simple extraction devices. The volume of the tips and their disposable nature make this approach attractive in the bioanalytical context. Several variables, including the number of dips, strokes, or sample volume, have been considered in detail to fully understand the potential of the sorptive phase. The combination of the microextraction technique and direct infusion MS allows the rapid detection and determination of three antidepressants (desipramine, amitriptyline, and mianserin) in urine samples with limits of detection in the ng/L range. Considering the chemical characteristics of the SWNHs, the new sorptive phase has the potential to interact with a great variety of compounds, especially those containing aromatic rings on their structures.

In this first approach a manual extraction is performed; therefore, the procedure is tedious and the sample flow rate cannot be efficiently controlled. As has been demonstrated, many strokes are required for the extraction, which indicates intermediate kinetics (the velocity of the extraction depends on the tip geometry as only a fraction of the aspirated volume meets the sorptive phase). In the same way, the synthesis (dip and evaporation) is done manually.

The potential automation of pipette tip extraction [41,42,43] or the use of static extraction procedures (where several samples can be extracted at the same time) may be a solution. Also, the synthesis can be automated by dip coating technology.

The stability of the coating is the main limitation for flow through applications, as the CNHs superstructure is not covalently bonded to the paper substrate. Also, assuring their stability in the paper-spray MS approach will be an exciting challenge in the near future.

## Figures and Tables

**Table 1:** Comparison of the sensitivity provided by the new method with other counterparts proposed for the determination of antidepressant drugs in biological samples.

Extraction Procedure ^1^	Instrumental Technique ^2^	Sample	Linear Range	LOD	Reference
Micro SPE	LC-UV	Urine	14–1000 µg/L	8.6–15.2 μg/L	[35]
Hollow fiber drop to drop microextraction	GC-MS	Water Urine Blood	0.5–50 mg/L	0.007–0.021 mg/L	[36]
Ionic liquid-dispersive liquid-liquid microextraction	LC-MS/MS	Blood	10–1000 µg/L	1–2 µg/L	[37]
Thin film microextraction	DCBI-MS	Plasma	5–1000 µg/L	0.3–1 µg/L	[38]
SPME	LC-UV	Urine	10–400 µg/	3–5 µg/	[39]
MEPS	GC-MS	Urine	0.1–100 µg/L	0.03–0.05 µg/L	[40]
Thin film microextraction	Direct infusion-MS	Urine	0.1–10	10 ng/L	This work

^1^ SPE, solid phase extraction; SPME, solid phase microextraction; MEPS, microextraction in packed sorbent. ^2^ LC, liquid chromatography; GC, gas chromatography; MS mass spectrometry; DCBI, desorption corona beam ionization; UV, ultraviolet detection.

## Supplementary Materials

The following are available online. Figure S1: Chemical structure of the four antidepressants drugs studied in this work. The logarithm of octanol/water partition coefficients (log P) for all the analytes at the working pH are also shown (source www.chemspider.com), Figure S2: Effect of the number of dips into the extraction recovery of the target analytes, Table S1: Selected reaction monitoring parameters for the MS analyses.
