# Supplementary material for: Carbon Nanohorn Suprastructures on a Paper Support as a Sorptive Phase
Source: Molecules. 2018 May 24;23(6):1252. doi: 10.3390/molecules23061252 (PMC6100432; doi:10.3390/molecules23061252)
Supplement: Supplementary file 1 [file molecules-23-01252-s001.pdf]

molecules

Article

Carbon Nanohorn Suprastructures on a Paper Support as a Sorptive Phase

Julia Ríos-Gómez, Beatriz Fresco-Cala, María Teresa García-Valverde, Rafael Lucena * and Soledad Cárdenas

Departamento de Química Analítica, Instituto Universitario de Investigación en Química Fina y Nanoquímica IUIQFN, Universidad de Córdoba, Campus de Rabanales, Edificio Marie Curie (anexo), E-14071 Córdoba, Spain; juliariosgomez@hotmail.com (J.R.-G.); q72frcab@uco.es (B.F.-C.); q72gavam@uco.es (M.T.G.-V.); scardenas@uco.es (S.C.)

* Correspondence: rafael.lucena@uco.es; Tel.: +34-957-211-066

Academic Editor: Victoria F. Samanidou
Received: 03 May 2018; Accepted: 22 May 2018; Published: 24 May 2018

Number of pages: 4

Figure S1 and S2

Table S1

1. UPLC-DAD analysis

Chromatographic analyses of antidepressants were carried out on a Waters AcquityTM Ultra Performance LC system (Waters Corp., Madrid, Spain) using an Acquity UPLC® BEH C18 column (1.7 µm, 2.1 mm × 100 mm) maintained at 40 ℃. The separation was performed under an isocratic gradient (30% B) using acetonitrile (solvent B) and water: acetic acid: triethylamine (0.1% acetic acid, 10 mM triethylamine) (solvent A) as mobile phase components. The system was re-equilibrated for 1 min between analyses, and therefore the total run time was 9 min. During the separation, the flow rate was maintained at 0.3 mL/min and 10 µL was injected with partial loop mode. The separated analytes were measured at 254 nm (mianserine and desipramine) and 252 nm (trimipramine and amitryptiline) using a PDA eλ Detector (Waters). System control was achieved with Empower software also from Waters.

2. Direct infusion MS analysis.

Table S1. Selected reaction monitoring parameters for the MS analyses

Analyte	Precursor ion ($[M+H]^+$ adduct) (m/z)	Product ions (m/z)	Fragmentor voltage (V)	Collision energy (V)	Collision cell accelerator voltage (CAV) (V)
Trimipramine	295.2	100.2 (Q)	125	20	7
		58.2		40	
Amitryptiline	278.2	233.1	140	20	7
		91.1 (Q)		40	
Desipramine	267.1	72.2 (Q)	145	15	7
		44.2		50	
Mianserine	265.2	208.1	135	20	7
		58.2 (Q)		30	

I.S	197.0	150.1 (Q)	125	25	1
		95.1		45	

Q: Quantitation transition

3. Chemical structure of the target analytes.

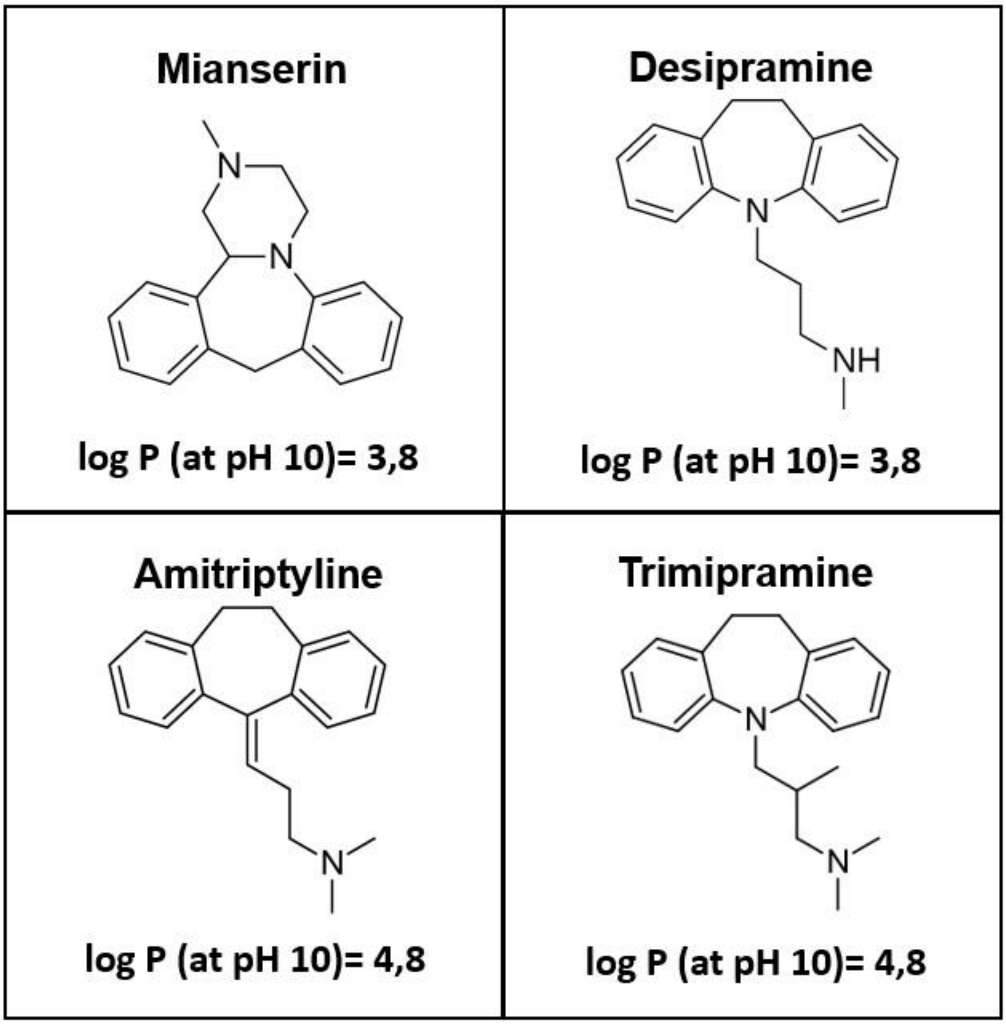

Figure S1. Chemical structure of the four antidepressants drugs studied in this work. The logarithm of octanol/water partition coefficients (log P) for all the analytes at the working pH are also shown (source www.chemspider.com)

4. Effect of the dips into the sorption capacity of the phase

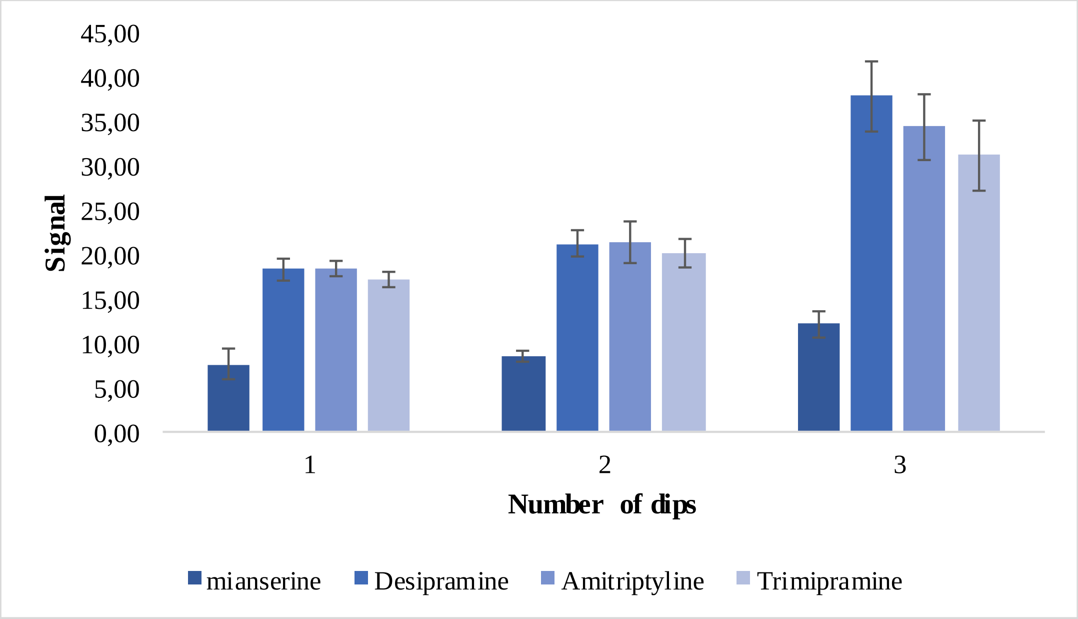

Figure S2. Effect of the number of dips into the extraction recovery of the target analytes.
